# Instrumental Technique, Expressivity, and Communication. A Qualitative Study on Learning Music in Individual and Collective Settings

**DOI:** 10.3389/fpsyg.2019.00737

**Published:** 2019-04-03

**Authors:** Andrea Schiavio, Dylan van der Schyff, Michele Biasutti, Nikki Moran, Richard Parncutt

**Affiliations:** ^1^Centre for Systematic Musicology, University of Graz, Graz, Austria; ^2^Faculty of Music, University of Oxford, Oxford, United Kingdom; ^3^Department of Philosophy, Sociology, Education and Applied Psychology, School of Human and Social Sciences and Cultural Heritage, University of Padova, Padova, Italy; ^4^Reid School of Music, The University of Edinburgh, Edinburgh, United Kingdom

**Keywords:** musical learning, embodiment, instrumental technique, expressivity, musical communication

## Abstract

In this paper, we present a qualitative study comparing individual and collective music pedagogies from the point of view of the learner. In doing so, we discuss how the theoretical tools of embodied cognitive science (ECS) can provide adequate resources to capture the main properties of both contexts. We begin by outlining the core principles of ECS, describing how it emerged in response to the information-processing approach to mind, which dominated the cognitive sciences for the latter half of the 20th century. We then consider the orientation offered by ECS and its relevance for music education. We do this by identifying overlapping principles between three tenets of ECS, and three aspects of pedagogical practice. This results in the categories of “instrumental technique,” “expressivity,” and “communication,” which we adopted to examine and categorize the data emerging from our study. In conclusion, we consider the results of our study in light of ECS, discussing what implications can emerge for concrete pedagogical practices in both individual and collective settings.

## Introduction

A growing number of contributions in music education research emphasize the value of pedagogical environments that involve close collaborations between music students and teachers, in which improvisation and shared musical activities are prioritized (Burnard and Murphy, 2013; Haddon and Burnard, 2017). Advocacy for such educational settings also comes from the scholarship concerned with the conceptual resources of the research paradigm known as “embodied cognitive science” (ECS) (Varela et al., 1991; Gallagher, 2005). This interdisciplinary elision has impacted both theoretical and practical pedagogical research (Juntunen and Westerlund, 2001; Bowman, 2004; Juntunen and Hyvönen, 2004; Borgo, 2005, 2007; Schiavio et al., 2018b), stimulating novel approaches that go beyond the traditional focus on individual skill acquisition and development (Burnard and Dragovic, 2014; Elliott and Silverman, 2015). Until relatively recently, Western music education tended focus almost exclusively on developing the technical skills and understandings required for the analysis and correct performance of composed works (Elliott, 1991, 1993; Lines, 2005a,b). Many have argued that this orientation downplays the creative potentials of students and teachers – reducing their status to mere reproducers of externally imposed criteria (the score, established modes of practice and performance, and so on), which often have little to do with their lives or their personal and collective histories (e.g., Regelski, 2012, 2016a,b; van der Schyff et al., 2016). As a result, new models are emerging that integrate the development of technical skills with open-ended forms of inquiry (improvisation, experimentation, the exploration of diverse musical cultures and practices, the creation of original music, etc.), intended to foster deeper awareness of the possibilities of musical learning as an artistic and creative process of self- and world-making. This includes educational settings that are based in collaboration and improvisatory musical interactions (Hargreaves et al., 2003; Biasutti, 2017; Nielsen et al., 2018). The purpose of this article is to explore more closely the relationship between such participatory forms of instrumental musical learning on the one hand, and – on the other – the theoretical tools central to ECS. The paper presents findings from a qualitative study based on music students’ reported experience of instrumental musical learning.

Following classic work by Varela et al. (1991), we define three main tenets of ECS as *sensorimotor coupling*, *bodily self-regulation*, and *intersubjective interaction*. From the outset, we map these three tenets onto three aspects of instrumental musical learning: *instrumental technique*, *expression*, and *communication*. Our decision to use these analogies is elaborated in Section “Finding the Body,” which argues for their phenomenological similarity according to published literature from music and the cognitive sciences. Additionally, these three aspects of technique, expression, and communication are uncontroversial categories to those concerned with music education. Each concept has its own discourse among practitioner communities, so learners are more likely to be able to verbalize about their experience with reference to these aspects.

We sought responses from (current or past) students who have experience of both one-to-one (student–teacher) learning, and also in group peer-to-peer ensemble performance classes. Discussion of both scenarios encouraged the respondents to reflect in a focused manner on the nature of the social interactions that present themselves in both formats of learning. This methodological approach elicited self-reported data that go beyond individual and practical challenges of musical learning, to include consideration of social-interactional factors. While we expected a number of important differences to emerge in the comparison between one-to-one and peer-to-peer ensemble performance classes, we argue that our data reveal some significant similarities. This suggests that – at least for the three categories considered – music students’ participation in music educational activities in both contexts is strongly mediated by immediate and imagined social relationships, and thus presents important continuities with more general concepts associated with ECS.

## Background

### Looking for the Mind

Cognitive science as we know it today can be traced back to the *cybernetics* movement that emerged in the early 1940s. It was during this period that researchers first introduced the idea that mental processes could be understood in terms of computations carried out by machines (Gardner, 1985). Such machines (a brain or computer) would consist of many simple threshold devices – i.e., neurons, silicon chips, or tubes that function in a binary on/off or active/inactive capacity – connected to be able to perform logical operations (Heims, 1980). In the early days of cybernetics, voices from the social and biological sciences held considerable influence and, because of this, there was much debate over whether such a strict computational model was sufficient to fully explain the functioning of the human mind. Nevertheless, the rapidly growing achievements of digital computing soon outshone alternative theories, and the computational model quickly became the dominant approach by which cognition was understood. By the mid-1950s, the field of cybernetics was largely focused on a clear computational hypothesis (Posner, 1989), with social and biological concerns shunted to the margins. The central idea was that intelligent behavior should be grounded in the capacity to *represent* the external world intentionally – that is, in the ability to cognize the “aboutness” of things and situations in the world. While this view may seem rather commonsensical, its laws and properties are not quite so intuitive.

Consider, for example, the question of how intentional states are physically instantiated, and how these states result in intelligent behavior – what then goes on between raw sensory inputs and intelligent behavioral outputs? The cognitivist answer is “symbolic computation” (see Bechtel et al., 1998, pp. 63–64): since symbols may be instantiated physically and may be ascribed semantic value, they can conceivably be subject to computational operations that function syntactically according to the language of the system (see Haugeland, 1981). Or, to put it another way, when semantic distinctions are encoded into the rules of syntax, abstract symbolic representations should become possible. These representations may then be logically manipulated (i.e., computed) by the system to produce further representations and intelligent output^1^. Of course, a computer carries out operations only on the physical form of the symbols available to it in accordance with the rules of syntax programmed into it by human beings. It possesses no knowledge of semantic values. The computer has no access to what a symbol, or group of symbols, is understood to represent, and therefore has no way of inferring the meanings of the computational processes themselves beyond the rules of its programmed syntax (Dreyfus, 1979). Nevertheless, the computer has provided the dominant model – or metaphor (Lakoff and Johnson, 1999) – for the mechanics, grammar, or “language” of thought. And thus, for most of its existence, cognitive science has focused on representations – the idea of syntactic operations on physical symbol systems – as the best way to understand the mind as an *inner* computer that processes information received from an objective *outer* reality.

This (inner/outer) conceptual distinction between mind and environment was not new. It is one of the central problems of modern philosophy, which apprehends the mind as the mirror of nature (Rorty, 1979). However, it is this very notion of the (rational-cognitive) mind as a disembodied category which poses several intractable issues for science of mind. In the first place, this notion draws the ontological relationship between mind and body into question, but it also raises epistemological questions regarding how, and to what degree, true objective knowledge of the world outside of our minds is possible. In line with this, a central aspect of the cognitivist model of mind is that the operations it describes must be played out at the preconscious or “sub-personal” level (Dennett, 1978): accordingly, we cannot be aware of such processes. In fact, we can *never* be aware of them. Solutions to address just how the representational outputs of information-processing mechanisms in the brain are meaningfully recognized by the system beyond the mechanics of syntax have led to “homunculus” metaphors and philosophical problems of infinite regress associated with an “inner theater” where “experience” is assumed to take place (see Churchland, 1983, 1985; Jackendoff, 1987). Such concerns notwithstanding, the idea that the musical mind should be, most fundamentally, a hierarchical, rule-based, representational, and “in-the-skull” phenomenon has until recently been a central assumption in music cognition studies (see Clarke, 2005; Moran, 2014).

In response to these dilemmas, the science of embodied cognition has sought a non-reductive perspective that sees mental life as dynamically distributed across the entire brain-body system in action (Thompson, 2007). In conceiving of bodily processes as *constitutive* of cognition – rather than as a distinct, supplementary apparatus – ECS maintains that no such strict separation exists between low-level and high-level domains of cognition – between unconscious physical actions, and apparently non-physical conscious planning or abstract thought. Examples can be found in how “gestures that accompany speech share the burden of communication with that speech” (Goldin-Meadow, 2003 p. 193), or in how bodily states, such as feeling tired, can shape the content of perceptual experience, as demonstrated by Proffitt et al. (2003). As we report in the next section, this approach can be understood through its implications for three categories of daily cognitive functioning: “sensorimotor coupling,” “bodily self-regulation,” and “intersubjective interaction” (Thompson, 2007), and can provide important insights to current research and theory in music education.

### Finding the Body

In the form of contributions, for example, by Chemero (2009) and Fuchs (2017), ECS has brought forth vital changes to the sciences of mind and (inter)subjectivity (see Shapiro, 2011). The change in scope and perspective has permitted study of our experienced world as an emerging property of the dynamical coupling between the animal’s physiology, its sensorimotor organization, and the environment in which it is situated. This shift has given voice to novel understandings of music performance and music education (Small, 1998; Reybrouck, 2014; Elliott and Silverman, 2015; Walton et al., 2015) advancing our comprehension of human musicality and musical experience (Reybrouck, 2006, 2012; Leman, 2007). Recent contributions have addressed questions around how the body participates in musical perception and emotion (see Reybrouck, 2010; Menin and Schiavio, 2012; Maes et al., 2014; Leman and Maes, 2016; Schiavio et al., 2017a), and how the body drives the acquisition and development of musical skills from early infancy (Gerson et al., 2015). For example, it has been argued that music perception is intrinsically linked to the motor expertise of the perceiver (Overy and Molnar-Szakacs, 2009), and that much of what is shared in musical settings involves bodily gestures, actions, and movements, rather than mindreading or other form of communication based on language (Schiavio and van der Schyff, 2016; Salice et al., 2019). Scholars inspired by this paradigm draw on several interconnected concepts, including aspects defined by Varela et al. (1991) as the “three dimensions of embodiment”: “sensorimotor coupling,” “bodily self-regulation,” and “intersubjective interaction.” In the following sections, we argue that these principles present important lines of continuity with three concepts associated with teaching and learning music: “instrumental technique,” “expressivity,” and “communication.”

#### Sensorimotor Coupling/Instrumental Technique

The first concept we wish to discuss is “sensorimotor coupling.” This refers to two mutually dependent aspects, which are biological and cognitively co-determined: (i) the processes of sensorimotor integration occurring at neural level and (ii) the patterns of action and perception enacted by a living system within its niche (see also Schiavio and Altenmüller, 2015). The complex sensorimotor connectivity of the brain flagged by (i) is a topic of considerable interest or the music cognition community, whose research has demonstrated that listening tasks may involve activation of neural circuits associated with action planning and execution, modulated by the (music-motor) expertise of the listener (Bangert and Altenmüller, 2003; Molnar-Szakacs and Overy, 2006). A corollary of such findings is that musical listening should be construed as an active form of musical engagement, rather than a passive or “disembodied” mental activity. The second aspect of “sensorimotor coupling” deals with the types of relationships that living systems enact with(in) their niche. Consider, for example, human infants’ early developmental capacity to manipulate objects of the environment through systematic explorations and motivated behaviors (Schiavio et al., 2017b). Such behavior involves a continuous integration of perceptual information and motor activity that dynamically determine each other; a rich interplay of action, perception, and (social) experience, culminating in the acquisition of new sensorimotor knowledge. More generally, the manipulation of objects in infancy can also facilitate the acquisition of communicative and linguistic abilities, highlighting the foundational role of action for a broad range of other skills. As Focaroli and Iverson (2017) remind us, the manual exploration of an object may stimulate the infant’s lexical development, enhancing “opportunities to extract information about object categories” (ibid., p. 22).

A similar focus on bodily skills and perceptual experience can be found in studies of the way in which musicians relate to their instrument – many of which are reported through ethnomusicological research (see Baily, 1995; Brinner, 1995; Rahaim, 2012). This topic is of particular interest in the context of music education, where novel musical meanings are formed and transformed through sustained practice. Here, we identify the area of *instrumental technique* (applying equally to vocal as well as to externally manipulated musical instruments) as a core factor which shapes the learning trajectory of a novice: in exploring new ways of musicking through their own instruments, learners develop important understandings of the musical world being enacted. Engaging in these kinds of activities can involve stressful and frustrating periods for students, as well as positive forms of motivation and feelings of accomplishment. Therefore, it is important to consider how students describe their experiences in one-to-one and collective settings so that we may better understand what kinds of interpersonal dynamics foster the most effective learning environments.

#### Bodily Self-Regulation/Expressivity

The second main dimension of the embodied approach – “bodily self-regulation” – concerns the intricate (metabolic, neural, chemical, affective, thermodynamic, etc.) patterns of bodily activity that serve to maintain the well-being of an organism and to preserve its status as auto-sufficient (see Maturana and Varela, 1980, 1984). Such biological networks describe *prima facie* sets of internal structural properties – processes that do not first appear to be constitutive of the coupling between the organism and the world around it, but are intrinsic to the individual. A closer look at the organizational laws of these processes, however, reveals that the internal biological norms of living systems are in fact in a dynamical relationship with their environment (Di Paolo et al., 2017). For an organism to flourish there must in fact be a corresponding, correlated domain (Thompson, 2007) – a socio-cultural niche, into which an organism (of any degree of complexity, from a bacterium, to a bat, to a barrister) can bring forth behaviors which reflect its own biological identity. We propose that a similar tension between personal and extra-personal dynamics may be found in the concept of *musical expressivity*. Expression “encompasses all changes in parameters that do not actually change the identity of the musical sequence. Expressive performance is also how performers display the deepest and most personal aspects of their work” (Lehmann et al., 2007, p. 85). By reflecting the musician’s perspective of a musical gesture – or the listeners’ inference of it through their own sphere of experience – expressivity is enacted in musicking as a “point of view” that permits participants to engage with, interpret, and transform, the musical ecology being created in a personal way. However, because individual contributions to musical environments are co-determined by social and cultural constraints, it is also important to consider how students experience learning and developing expressive skills in one-to-one and collective settings.

#### Intersubjective Interaction/Communication

The final dimension of embodiment, “intersubjective interaction,” refers to the pragmatic notion that processes of social understanding are intrinsically dependent on bodily activity (De Jaegher and Di Paolo, 2007). By this view, direct forms of reciprocal interaction and participation are seen to constitute the enabling condition of communication – a statement which appears to describe *de facto* the situation in which all forms of communicative systems and behavior arise and develop. Yet, ECS’s foregrounding of this issue is crucial: in short, communication as *intersubjective interaction* can only be achieved through participation in acts of relating and the enactment of social relationships, and not as the consequence of a prior process of mindreading (Schiavio and De Jaegher, 2017). The argument is therefore that body and action can reveal much about ourselves and others, without necessarily involving inferential processes of mental states’ attribution (see Schiavio and Høffding, 2015; van der Schyff and Krueger, 2019). Theorists of ECS tend to frame the discussion on intersubjectivity by highlighting the continuity of so-called top-down and bottom-up processes in social cognition, drawing attention to the ongoing negotiation between objective and subjective aspects of lived experience and the mutuality of action and perception in any intersubjective context (Thompson, 2007). Musical contexts present an ideal domain to capture such forms of direct understanding: studies of musicians’ communication in rehearsal and co-performance now spans various contexts (e.g., Turino, 2008; Moran, 2013; Timmers et al., 2014; Loaiza, 2016), and has given rise to novel understandings of non-verbal effective communication in skilled contexts. The category of *communication* stands in for an instantiation of intersubjective interaction in the musical domain.

In what follows, we report on our qualitative study, which involves questionnaires using open-ended questions with 19 participants who currently study or have studied music in different contexts. In doing so, we aim to clarify how the three categories reported in this section are described and experienced in individual and collective pedagogical settings.

## Materials and Methods

The present study is part of a larger project examining how music teachers and music students experience individual and collective pedagogical settings. Ethical approval for the study was granted by the Ethics Committee of University of Graz in December 2017, for the recruitment of questionnaire respondents. The questionnaires were designed to capture a range of responses, thoughts, beliefs, and practices. The present study focuses on the analysis of data according to themes related to instrumental technique, expressivity, and communication.

### Participants

Recruitment of participants from the target population of instrumental music students took place through researchers’ own networks, social media, and advertisement at local music schools. Initial email exchanges were used to assess participants’ suitability according to the following inclusion criteria: having at least 5 years of musical learning experience and being 18 or older. Those who met these criteria were asked to read and complete (i) a consent form which explained the purpose of the study and subsequent use of data and (ii) a questionnaire (electronic format). A total of 19 students from music schools, conservatoires, and universities in both Europe and United States took part in the study (12 females, six males, and one participant who identified as non-binary). Their age spanned between 20 and 36 years old, median 28.5 years old. Participants’ musical backgrounds varied; all had attended individual and/or collective instrumental lessons for at least 5 years (range: 5–30; median = 13.5), playing one main instrument: piano (*n* = 5); violin (*n* = 4); guitar (*n* = 2); harp (*n* = 1); drums (*n* = 1); oboe (*n* = 1); sitar (*n* = 1); flute (*n* = 1); trombone (*n* = 1); bass guitar (*n* = 1); and voice (*n* = 1). Participants did not receive any payment or financial reward.

### Materials

Most participants (*n* = 16) received an open-ended questionnaire via email. This comprised a background section to collect demographic and prior musical experience data. The second section posed general questions concerning teaching and learning. The questionnaire was originally designed by four of the present authors (AS, DvdS, MB, and RP) for a prior study within the larger project (Schiavio et al., 2018a). In this prior study, the open-ended questions were used to explore themes related to the teachers’ experience of “presence” during individual and collective lesson. The total of 18 items on the questionnaire deal with different aspects of individual and collective musical classes. Examples of questions include: “How is theoretical knowledge taught in individual and collective classes?”; “What are the collaborative, interactive, or ‘relational’ aspects you like the most in individual teaching settings?.” The final three participants to be recruited to the present study received an adaptation of the original questionnaire, comprising 13 items. Based on the review of existing questionnaire responses, this iteration allowed the authors to further expand on the original set of questions (see Agee, 2009). Because the data were already quite rich, this final set of participants was considered to be enough for the purpose of the study. Examples of questions include: “How could expressivity improve in individual classes?” and “How important is it to focus directly on instrumental practice when participating in collective classes?” Participants were instructed to respond fully and discursively to each question, without word limits; to include details even if they thought them trivial; and were encouraged to provide concrete examples where possible. In four cases, participants were contacted by the research team to better clarify certain ambiguous statements and to elaborate on aspects relevant to the present study. In these cases, short semi-structured interviews were audio-recorded and transcribed. The duration of these interviews varied between 15–20 min.

### Data Analysis

The content of participants’ responses was examined and categorized according to the three pre-determined categories:

• Instrumental technique
• Expressivity
• Communication

**FIGURE 1:**
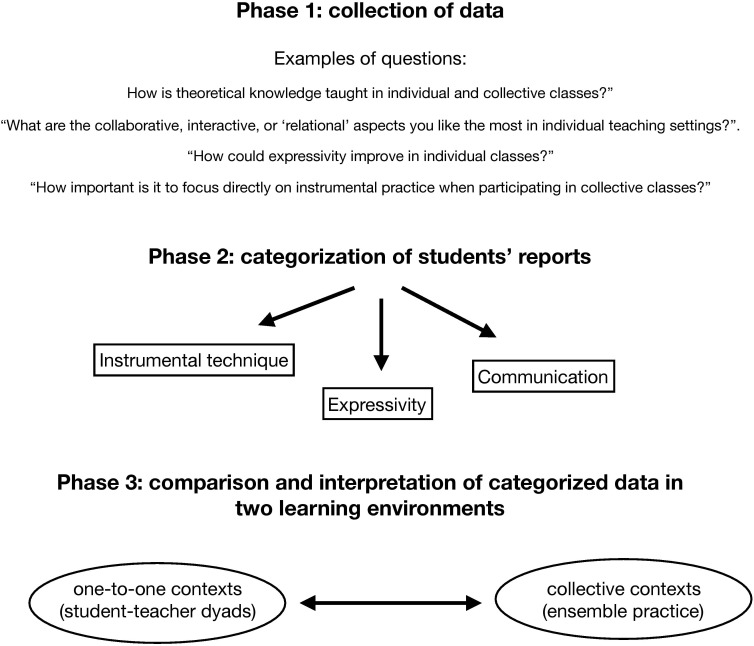
The three phases of the coding process.

These categories were developed on the basis of our discussion in Section “Finding the Body.” Similar theory-driven approaches were successfully implemented in qualitative research, resulting in a number of cross-disciplinary publications (see Schreier, 2012). The coding process adopted here involved three main steps: (i) preparing the ground of the analysis and becoming familiar with the raw material; (ii) categorization of the data; and (iii) interpretation. Following the initial conceptual organization of the themes, the *first phase* (i) involved initial immersion by AS and MB, in which all primary data were read several times. This work involved assessment of the participants’ experience of instrumental technique, expressivity and interpretation, and communication. The data were identified, segmented and re-organized (e.g., translated into English) in preparation for the next stage of analysis. This *second phase* (ii) centered on the categorization of the participants’ answers. Here, our pre-existing codes were used to classify the content of each segment. This step involved a novel level of categorization, which was adopted to distinguish between participants’ descriptions of individual versus collective musical settings. This yielded a selection of ordered data, which retained direct quotation of participants’ own responses. The final selected data were checked to avoid repetition, and were subsequently verified by AS, NM, and DvdS. In the *final phase* of the process (iii), the researchers explored possible interpretations of the data. All quotations were then carefully re-examined, and the report of the study was discussed. The three phases are reported in Figure 1.

## Results

### One-to-One Lessons

In this section, descriptions of experiences associated with individual learning are reported. Particular attention is devoted to reporting the learners’ own verbal accounts and to highlighting examples in these accounts that are most characteristic of their learning experiences.

#### Instrumental Technique

Practicing and developing a meaningful relationship with a musical instrument represent two intrinsically related aspects of learning instrumental technique. These include the student’s relationship with an instrument and with a teacher, respectively. The dynamical integration of these material and social aspects involves different ways to experience the sensorimotor dynamics of musicking. For example, as one of our participant reports, it is important to:

“Feel a good sense of connection from my body to the instrument […]. [F]eeling the instrument as an extension of your own body – or, if you like, breaking down the boundary between body and instrument – may be advantageous.”

From the outset, this highlights the primacy of the body and its physical interaction with instrument – where ideally, the boundary between the two becomes transparent. However, the process of achieving this extension of the body into the instrument (or incorporation of the instrument into the body), can be highly uncomfortable and frustrating. Here the learner is required to explore and develop novel patterns of motor action as different bodily configurations are enacted. Initially, the results may be disappointing, leaving the learners in a somewhat awkward relationship with their own corporality. Because of this, it is helpful to have the guidance of another who has experienced such processes. In the context of individual learning, then, connections are extended beyond the student/instrument relationship to include the role of the instructor and his or her relational approach to teaching. Consider the following two statements:

“Training directly with your teacher is the best way to learn: he/she can guide you through technical [advice] and [give] suggestions”

“I feel comfortable when the teacher is knowledgeable, knows both the technique and how to approach the music. Also, when the teacher does not waste too much time in individual specific problems but teaches general principles and concepts that then I can apply on my own to solve other issues.”

Engaging in musicking with the teacher is a fundamental aspect of learning instrumental technique. This can be done in different ways: by performing together, by encouraging students to explore more solutions for a specific technical problem, or by cooperatively discussing other possibilities and options. These options are described in the following two quotes:

“Often the aspects that are most uncomfortable, like learning or improving technique, are most effective contributors to development. I also believe improvising with and imitating the instructor to be essential to my development. Specifically playing drums together with my instructor, trading fours or eights while practicing a groove was really helpful [for] solidifying a musical concept or feel in my head.”

“While studying I focused on technique, specifically as it applied to repertoire or desired learning outcomes. In the jazz curriculum there were technique and repertoire requirements and often the lessons focused on best practices and instruction on the meeting those requirements. Often, we would listen to and discuss recordings of note for examples of what I was working on. Occasionally when working on grooves my teacher and I would take turns improvising.”

When the relational aspect between teacher and learner does not function adequately, however, the student’s experience may be significantly impacted:

“I don’t like it when my teacher states that there is only one way to fix things or one way to play something. Practicing/performing an instrument cannot be just black and white […]. Very few of my teachers have showed me how to practice efficiently; I’ve had to do my own research to find that out.”

As a result, this can lead to situations that might be stressful:

“Some days I think that there is definite improvement, other days not so much. I keep goals in mind and I tell myself that as long as I stick to these goals and attain them, then I can move on to the next set.”

In referring to such situations, participants mentioned also some strategies to control and to cope with stress. Consider the following quote:

“I feel a great deal of affection for my last teacher, she understood I did not aim to become a professional pianist but instead helped me define what kind of pianist I wanted to be. After deciding I would not become a professional pianist the teaching process stopped being stressful at all. Instead it became the most relaxing aspect of my life. Before then, it was mildly stressful, but only because I was also doing high school at the same time.”

These statements show that the bodily engagement in musical learning involves more than developing fluid interactions with an instrument. It also includes a range of emotional-affective and empathic dimensions that must be attended to if effective learning is to occur. For example, consider how the last statement provides evidence that mutual understanding with the teacher can help in alleviating and controlling the experience of stress. Students reported that they experience less stress when forms of engagement emerge that are driven by motivations and understandings that are shared with teachers. But is this something that only refers to the acquisition of technical skills or does it involve other musical dimensions?

#### Expressivity

As we saw, the focus on instrumental technique is not separate from how students meaningfully interact with their teachers and their instrument. As we also considered, corporeal, emotional, and empathic factors are central to such relationships. As these couplings evolve, students develop a personal musical identity that is brought forth in each performative situation. This involves the development of a repertoire of expressive devices and understandings that allow them to make unique creative and interpretative contributions to the musical environments they participate in. Despite the intrinsically personal and subjective characteristics of expressivity, its relational aspects become important in learning. Consider the following quote:

“I liked most the collaborative approach my last teacher took when we worked on interpretational choices. In the end she said I influenced her as well in her interpretational choices.”

A similar point is made by another participant:

“I love to collaborate with my teacher and I love when he listens and values my ideas and creativity […]. I don’t like when teachers have an old-fashioned way of teaching in which they only want the student to repeat their interpretation of the piece, without acknowledging their [(the student’s)] own musicality.”

This suggests that expressivity is not a property that is best applied after a piece is (“mechanically”) learned. Instead, it is an important component of the learning process itself, which cannot be entirely separated from other aspects (see McPherson and Gabrielsson, 2002). If expressivity is indeed continuous with different emotional, social, and technical features of the music, then it follows, as this student suggests, that the best way to learn a piece might involve:

“Incorporating expressivity early in the learning of a piece, rather than focusing solely on technique first.”

Consider now the following two quotes where this dimension is discussed in continuity with affect:

“I think expressivity can be cultivated through (1) being allowed to play music of your choice e.g., classical composers that are most admired by the student and (2) by a teacher who is emotionally engaged and can convey their experience more readily and inspire the student.”

“Expressivity and emotional engagement can foster instrumental technique much more efficiently than simply studying/repeating a piece of music.”

Additionally, because of the highly affective nature of musical development, it may be disappointing when teachers have different opinions about one’s interpretative choices. As one participant reports:

“I feel uncomfortable when the teacher criticizes my expression of the music.”

A final example can further clarify this point:

“As I was about to do an important violin exam, my teacher and I worked really hard on expressive gesture, rather than more technical aspects of the instrument like, say, scales or fingering. She says that for example how you start your performance is really important and will definitely influence the judgment that a committee or an audience will develop toward you. Not only expressiveness translates in a different kind of impulse in the way you perform, but also at a visual level it provides a deeper layer of understanding for the audience. It creates connection and allows them (the audience) to get to know you better. Students do not really focus on this aspect at the beginning, as you have 5000 other worries you have to take care of. But my teacher, in fact, was right – you should not take this aspect for granted.”

Not only does expressivity reflect personal choices in performance; it also creates an opportunity to explore the different affective and technical nuances of a piece. However, the last two quotes highlight the vulnerability and pressure felt by some students, indicating that teachers should be thoughtful about how they offer criticism. Ideally, and as the earlier quotes suggests, critical feedback from the teacher is most effective when balanced by an empathic awareness of the student’s own interests and creative goals. This helps the learners develop their own expressive possibilities in continuity with insights from the teacher and relation to the contextual norms in which the piece (or musical practice) is learned. Additionally, and as the last quote suggests, the bodily movements associated with musical expressivity have an important influence on the experiences of performers, audience members, and co-performers. This bodily dimension should be explored and conceived of as an important intersecting point between technique and expressivity. This brings us to the topic of communication, which can help us better capture how such features are negotiated and mutually understood by students and teachers.

#### Communication

As we have seen, both technique and expressivity have strong roots in corporeal, emotional, and empathic relationality. With this in mind, let us now explore in more detail what students report about their communication with teachers. As we will see, several interacting aspects are involved in building effective communication in the context of instrumental learning. Consider the words of this participant, who reflects on what he feels is the main aim of an instrumental lesson:

“The purpose of the lesson is usually to improve the musical skills of the student, so in some way the student is communicating their musical skill and their musical ability to the teacher, who is listening attentively for ways to improve. Sometimes a teacher will then play what they want the student to do, so teaching/communicating through music.”

As expected, this sense of connection builds through musicking and open discussion. Consider the following two quotes, where both aspects are reported to play a key role:

“Imitating and improvising with my teacher was the most enjoyable. It is a rarity to get to learn from a master at my instrument, and so playing alongside someone with that amount of skill and knowledge is great. I also really enjoyed conversation about recordings or music in general. Those helped build rapport and were a great way of expanding my understanding of music.”

“I feel very comfortable when my Professor is willing to discuss eventual different opinions and provides several argumentations for the instructions he is giving me.”

This leads to situations where both students and teachers may learn together:

[I like it] “when a teacher recognizes how the student “is ticking” and both can learn from each other. [This can happen] when communication between teacher and student is working (taking each other seriously, appreciation).”

Students mentioned also some obstacles that could interfere with communication. For example:

“The “imprinting” factor is really important in individual classes, and it’s impossible for a teacher to avoid creating students which play in a very similar way; though, it would be good for a teacher to try to only pass to the student its knowledge, without influencing its style too much.”

Most importantly, how teachers and students communicate has repercussions for both technical and expressive aspects.

[I would like to improve] “how to better communicate the most nuanced parts of performance technique and expression. How can we talk about the detailed execution of ornaments, phrasings, subtle timing; these details in the teachers playing are difficult to discuss in language and often hard to just ‘do’ at the instrument.”

It thus seems that no clear boundaries exist in individual music lessons between the categories of instrumental technique, expressivity, and communication. By analogy, it may be argued that the relational dynamics between students and teachers in individual settings encompass all three dimensions of ECS – again, *sensorimotor coupling*, *bodily self-regulation*, and *intersubjective interaction*. Importantly, these dimensions do not describe discrete aspects of the communicative processes in such contexts. Rather, they are in constant negotiation as different forms of communication and reflection emerge adaptively as the lesson unfolds. These involve the rich network of empathic, affective, comparative-mimetic, verbal, bodily, demonstrative, and analytical processes involved in the participatory cycles of communication and reflection enacted by both agents. In line with the insights of ECS, this highlights the inseparable continuity between bodily, affective, and cognitive processes (Colombetti, 2014; Schiavio et al., 2017b). It also gives rise to important questions concerning whether the same interactive necessities are developed when the pedagogical environment involves a larger class size. Are similar relational learning dynamics extended to other students? Or are they more closely associated with their teachers? And how do students motivate themselves and learn novel skills when other peers are involved?

### Collective Lessons

In this section, we report excerpts from music students concerning how instrumental technique, expressivity, and communication, are developed and experienced in collective pedagogical settings. These mostly involve music classes where students play different instruments (e.g., orchestra), but also cases where peers study the same instrument (e.g., piano or guitar duos). In both cases, we have one teacher and two or more students. As we will see, differences in number of participants in collective learning situations does not have as much impact on the reported experience as one might expect.

#### Instrumental Technique

We found that students who participated in our study exhibited different attitudes and perspectives when asked about instrumental technique in collective settings. However, one common theme that emerged involved the important role played by other students:

“The instructors are mostly there to provide criticism on the performance, and typically not on individual technique. In small ensemble courses I took, often the instructor would only be present for a few minutes each rehearsal, rotating between the other ensembles in the class, and then be present for the performance evaluation. This meant that a lot of minor correction and revision happens between peers, which I enjoy. Many things, like informing me that I was dragging in the bridge, or that we should bring the dynamic down on the second chorus, etc., could be brought up by peers rather than the instructor.”

Peer-to-peer learning appears to be one of the core aspects of the collective lesson, and a way for improving the technical level with the help of the other colleagues. Unsurprisingly, the sensorimotor couplings enacted within collective contexts seem less determined, and less focused on a single (e.g., technical) aspect of it. Instead, they are much more open to the contextual demands of the lesson:

“In my program there were ensemble classes that were focused on learning tunes and putting together performances, often in a matter of a few rehearsals. What I really enjoyed about those was the collaboration from all band members that was required in order to have efficient rehearsals that yielded a good final product. In many cases it was a matter of speed-arranging tunes, so everyone was able to contribute ideas for how to best arrange the material to optimize the final performance.”

This quote highlights the insight that the final result is a product of the group, with its global features being determined by reciprocally active contributions. This implies that the sensorimotor couplings between agents and the (musical) environment developed in collective settings involve a shift in focus from the instrument to the group. This contributes to creating a collaborative environment where all the students are focused on the moment-to-moment features of musicking. Consider the following three quotes, from three different students:

“The other thing that I really valued about group classes was it was one of the only times in jazz school that I felt like I was actually ‘doing the thing’ that we were all there to study. No textbooks, few requirements, just music-making with a professor to offer suggestions and to critique our playing.”

“I like the repertoire that you can play with others. I like responding to each other’s impulses.”

“Chamber music lessons were great. Again, when you like your partner, it is great when you have the same musical intention and want to realize it when playing.”

The last quote also highlights well the contextual and affective-empathic couplings that are enacted in the process of musicking, where social agents participate and integrate their own sensorimotor agency. However, this might involve some problematic issues as well. As one participant noted:

“There is shared responsibility in the collective lessons, and the teacher doesn’t only focus on me, so I care much less about the quality of my work.”

While it seems that instructors in collective settings are less involved in the individual acquisition of technical skills, students can still improve through mutual assessments and collective musicking. The feeling of a good connection with the musical instrument reported in individual contexts is here translated into a relational and empathic property that is necessary to create a cooperative learning environment. In both cases, learning is never fully experienced as a solipsistic reception of external stimuli, nor it is decoupled from the socio-material environment in which the learner is embedded. As we saw in Section “One-to-One Lessons,” moreover, instrumental technique is also continuous with expressivity. Let us see what happens, then, when students are asked to discuss their experience of expressivity in the context of collective lessons.

#### Expressivity

Differently from individual settings, participants reported that it may be difficult to combine expressive and technical skills in collective classes:

“I feel the few collective classes I have been involved in [have] focused on expressivity and instrumental technique separately – the teacher identifies an issue and figures out if that issue is an expressive issue or instrumental issue and responds accordingly in their teaching.”

“It is difficult for the teacher to find a good balance between all the students. There are different levels of preparation and expressiveness can be difficult to be learnt this way. Also, advanced students might be less motivated, and novices more stressed when put together in the same class.”

This initial difficulty, however, might be mitigated by some of the performative aspects of the group lesson, which, among others allow for the development of new forms of bodily awareness:

“I tend to move a lot more and am aware of my movements when I’m in collective lessons. I’m not exactly sure why, though – perhaps it’s from something my youth orchestra conductor stressed a lot – that you should always be leading, even if you are the last player in your section.”

This recalls the points made above regarding the foundational role played by the body in musical communication. The tension between the “point of view” of each student, and the emerging relational dynamics of the class is also well described here. While it may be difficult for the teacher alone to find the right balance between technique and expressivity – and by themselves facilitate their reciprocal co-determination in a group context – a student can find a helpful resource in the ways peers support and influence her or his own musicking:

“Being surrounded by people who also appreciate and care about the oboe is a really great feeling, especially because the instrument is not so popular. My peers especially understand the quirks and hardships that come with playing oboe, and I find a lot of comfort in the fact that I am able to spend structured time with them.”

And, again, when certain participatory and empathic dynamics are encouraged in collective situations stressful situations can be avoided:

“Consider this: if I learn my part at home, I come to class, and perform it perfectly – like a ‘clock’ – I am actually playing for me only. I do feel the difference when everyone is learning their own part together, as each of us is exposed to the ideas of the others. In such context, I can even anticipate the others’ parts when I perform mine. I have less stress because my own part is built on the others’… but that is not like they are covering me, or hiding my mistakes. Rather, I feel I am part of something new, where our expressive visions (‘styles’?) blend together. Not sure, though. Others really enjoy the feeling of being out there by themselves.”

This is echoed by another student:

“During collective lessons I feel more confident, comfortable and relaxed, as I feel almost backed by my colleagues.”

While at first glance expressivity in collective contexts appears to be a difficult area for instructors to convey, it can emerge spontaneously within the relational dynamics of the lesson, provided the collective learning environment is a positive one. Here the tension between internal and external points of view may be best understood by considering the single learner as a part of a network – an autonomous agent who reciprocally interacts with his or her peers; and who, in doing so, simultaneously enacts his or her own musical identity, and contributes to the collective identity of the group. The shifting demands of the music being created become fluid and flexible so that expressive components emerge and develop as the group behaves as a whole. But then, we may ask, what kind of communicative aspects are involved in such situations?

#### Communication

Clearly, communication plays a key role in how collective lessons unfold. However, the dynamics of this need to be better understood. For example, it would be interesting to know whether in such settings students are inclined to focus more on communication with peers or with the teacher. An initial impression might lead us think the former option is privileged.

“I like playing with others because there is a collaborative and empathetic aspect to it. I like building rapport with fellow musicians.”

“As long as it is comprised of people whom I like, there is a great sense of belonging and friendship when we create music together. However, those feelings aren’t present when that isn’t the case. I can sense feelings from other people playing, and if they aren’t happy with someone in the group, it’s noticeable. Egos need to be set aside!”

Note how the focus of these quotes involves emotional and empathic aspects central to social musicking rather than verbal communication. Here the negotiation between individual and collective subjectivities develops as the music unfolds: by playing together relational dimensions are developed, which involve an affective and expressive dimension. This helps students build mutual trust, enabling possibilities for open communication:

“Like in individual lessons, the emotional-affective sensations I felt in collective lessons depended heavily on my perception of how the lesson was going, as well as on the inter-personal dynamics of the group. When things were going well and we were having productive rehearsals and getting positive feedback from the instructor, I felt very content, proud of our work, and a strong bond with the other students in the group. When things were not going well, especially inter-personally (i.e., someone is wasting time on minutiae, late to rehearsal, argumentative), I would become anxious or frustrated, sometimes emotionally detached and withdrawn from the group, much to the detriment of the music. These are largely dependent on my own perception, and so can be improved by me re-evaluating the scenario and mentally re-framing any issues I’m having. For example, if I’m annoyed with someone for wasting the group’s time by being unprepared, I can mentally ease up a bit by remembering times I, too, was unprepared and how other people gave me the time I needed and it didn’t end up affecting the final product in a substantial way.”

While most of focus is dedicated to how others actively participate in the musical dynamics of the lesson, communication also depends on the personal attitude of the learner, who can creatively decide how involved he or she could be:

“In a collective lesson one has the ability to kind of choose how much or how little they are going to participate/contribute. This can be positive or negative, in ensembles I’ve been in with a healthy group dynamic, usually the push and pull of ideas is a good thing. Certain people sort of ‘champion’ certain tunes to lead/arrange and the rest of the ensemble can pull back a little bit and let them lead. In other, less balanced small ensembles I’ve played in, that sort of push and pull dynamic is absent, and typically one or two people end up leading the ensemble leaving the other members to feel creatively disconnected from the music they’re playing, resulting in a weaker final product.”

We should not forget that the educator is also part of the group the learner is interacting with. However, it seems that the teacher cannot impose a particular strategy to develop effective communicative dynamics. Instead, it seems that communication self-organizes itself as the lesson unfolds:

“Interaction with the teachers or with the other students is not taught. My teacher, for example, was always trying to give us instructions about how to interact: ‘if you have to start, do a specific gesture with the right intention and make sure everyone is looking at you.’ He was asking us to gain coordination between ourselves by looking at us or by carefully listening what we were playing. He was very demanding, and this type of oral communication did not really help.”

This implies that teachers should be cautious of imposing contexts that are too prescriptive. Rather, they need foster learning environments that encourage students to develop and share their own insights relevant to the communicative aspects of (collective) music-making. Consider the following quote, where an example of this is given:

“Recently I have noticed how some teachers try to stimulate the interactive dimension among students outside of the classroom. Some asked us to get together and study together, implying that we should not [only] perform, but also discuss the piece, and mutually learn from each other. Then, of course, it depends on the student’s sensitivity, but obviously I did enjoy this dimension where you can communicate with a peer who is at your level. And this can stimulate and help shy students in particular.”

In collective musical settings, we observe very similar dynamics to those that emerged in individual settings. Data concerning our three categories present overlapping characteristics that describe how positive environments for collective lessons (which encourage collaboration and self-reflection) afford healthy opportunities for students to participate in each other’s learning. Although their focus shifts here from their instrument (and their teacher) to the whole group, the fluid integration of technique, expressivity, and communication suggests that while different strategies and social dynamics are enacted, similar aims and challenges are involved in both contexts. As we consider next, developing better understandings of the differences and continuities between individual and collective learning contexts could lead to more effective pedagogical environments where these aspects of musical learning are integrated more effectively. This may include the development of novel teaching strategies that emphasize the reflective, verbal, and relational dimension of the students’ learning dynamics, encourage the development of shared learning goals and trajectories, and inspire students to actively seek collaborative experiences beyond the lesson.

## Discussion and Conclusion

Our data offer considerable insights into the individual and relational dynamics of instrumental music classes from the point of view of the students. Within such contexts, our findings emphasize the key role of body and (inter)action in determining and transforming the learning trajectory of each pupil. In accordance with the main tenets of ECS, the categories of “instrumental technique,” “expressivity,” and “communication,” are here understood as relational: regardless of the different pedagogical settings in which they emerge and develop, they are realized through their reciprocal interaction. Indeed, while individual and collective pedagogical settings display a number of structural and organizational differences, they also share important dynamics concerning how these categories mutually determine each other.

Whilst traditionally associated with rigorous instruction and individual practice, the development of “instrumental technique” is shown here to be a more flexible and fluid phenomenon. In individual (one-to-one, student-instructor) learning contexts, this asks us to look more closely at the how the relationships between student, instrument, and teacher affect musical development – physical and social environments play a key role in the acquisition of the technical skills required to perform at an optimal level. If development were associated only with the input of teacher, then it also seems unlikely that a similar account would emerge in collective settings, where the main focus is on the ensemble. Instead, we noticed how similar relational dynamics are also present in these environments, though they play out in a different social arrangement. These dynamics are here modulated through the effort of the entire group rather than only through interaction with a teacher, giving rise to novel patterns of action and social configurations that emerge within the collective musicking. The shift from individual to collective contexts, however, is not unproblematic: it presents important challenges for the unfolding dynamics of the lessons and for the student’s own learning. Consider the following quote:

“In individual lessons there is more room to face your personal technical challenges. Because, if for some reason a passage does not play out ok in a collective class, I can always change fingering without my teacher noticing. That’s because achieving a good collective sound is more important than everything else, in terms of instrumental technique. Instead, in individual classes I have to be much more prepared technically. There is no one I can hide behind.”

This quote is interesting because it conflates the notion of instrumental technique with a pre-defined motor program that is developed and instantiated in accordance with the teacher. However, the ability to change fingering and adapt to the changing demands of the group can be arguably considered as a sophisticated form of instrumental technique – one that allows the learner to creatively adapt to the moment-to-moment contingencies of the music being performed. And because, as another student reports, [in both individual and collective lessons] “your interaction with your own instrument is the same,” we find that differences concerning the acquisition of technical skills depend highly on the relational properties of the pedagogical setting in which one is situated. Where teachers might be seen to foster a more prescribed form of technique, peers can help stimulate novel actions relevant to the contingent musical opportunities afforded by the emergent sonic environment.

Similar differences were noted with regard to the notion of “expressivity.” In general, most students agree that this is not easily detachable from the concrete patterns of action necessary to play an instrument. To capture the idea with an example, even the “mechanical” repetition of a scale need not be considered “un-expressive.” Instead, there is a sense in which it is *inherently* expressive – as one plays the scale, the bodily effort and ancillary movements adapt to and develop in relation to the shifting tones and intervals in a meaningful way – one that will involve affective dimensions if only subtly (e.g., as one moves from the third to the fifth and then to the octave). The point here is that actions and emotional connotations cannot be easily detached from each other (see Colombetti, 2014). As such, expressive musicking appears to be a constitutive aspect of learning technique, rather than a category to be superimposed when an adequate technical level is achieved. While it is perfectly reasonable to think that the relationship between technique and expressivity could be a focal point in individual teaching settings (e.g., a teacher could help the student focus on such aspects while doing specific technique-related exercises), it might be difficult advance in collective settings. Here the focus on the individual is traded for a more general focus on the musical cohesion of the ensemble. As two students reported:

“In individual lessons, I can work on the precise problems that I’m having. In collective lessons, we have to focus on the precise problems as a group as a whole.”

“In individual lesson, I am able to focus more on my own technique as well as repertoire I am preparing – there is time to dive into more specific questions and concerns I have. When we perform for studio, we address bigger musical concerns, performance strategies, and also discuss professional musician etiquette.”

As we saw, however, these differences do not downplay the musical development of the individual within the collectivity. One is not simply “absorbed” within the ensemble. Each learner displays layers of expressive autonomy that are enacted in musicking, though modulated and negotiated within the intrinsic demands of the group. For instance, one can enjoy the others’ comments, feedback, and suggestions, and develop novel expressive features that could enrich the group performance as well as address specific technical difficulties that are relevant for his or her own learning. Using different fingerings, phrasing, or dynamics, can be highly functional for both collective and the individual levels, leading to novel expressive and technical resources. This means that effective musical development in these contexts is also dependent on fostering a heathy relational dimension, where individuality and collectivity flourish as a dynamical system. This provides the student with the opportunity to reframe specific aspects of his or her musicking and critically engage with them in an adaptive context.

In line with this, the dimension of ‘communication’ was investigated across both pedagogical settings. In individual contexts we saw that optimal communicative experience between students and teachers provides the former with important resources that are brought forth in the act of musicking. This results in a more efficient understanding of the interplay between expressive and technical elements – verbal and behavioral aspects of learning are integrated and negotiated as the lesson unfolds, just like technical and expressive aspects co-constitute a structural unity. The facilitating role that such open forms of communication play can be also observed in collective settings. However, here the more direct interplay between teachers and students is traded for the complexity of group dynamics. Because of this, instructors may need to employ simpler or more general communicative strategies to help the ensemble achieve certain musical goals. Indeed, in such contexts, detailed discussions involving the relationship between technical and expressive dimensions might not be helpful or possible. Here the teacher may focus instead on identifying broader concerns that help to clarify the shared goals of the ensemble. In collective settings, as one student reported:

“The focus is not only on you. That is, typically more focus on interpretation than on technical matters. Often the teacher will not be that familiar with the technical aspects of your particular instrument. Sometimes the goal is the same, but the teacher will communicate it in a different way because they cannot say “do this and that with your arms/hand/wrists,” but rather have to say “make it sound like this or that.”

It should be noted that this does not imply that technical and expressive aspects become separate dimensions when performing and rehearsing in collective situations. As we saw above, positing a strict distinction between them remains a highly artificial move that cannot capture the dialectics between a person’s musical identity and his or her musicking. And indeed, the expert teacher is well aware that technique and expressivity can be realized through other means – without a strict focus on each of them. For example, when the broader set of musical “goals” is collectively engaged with, a larger horizon of musical challenges and opportunities arises, which facilitates the emergence of novel skills. Here musical actions may be developed and meaningfully explored between the members of the ensemble themselves – and often with little or no intervention from the instructor.

**FIGURE 2:**
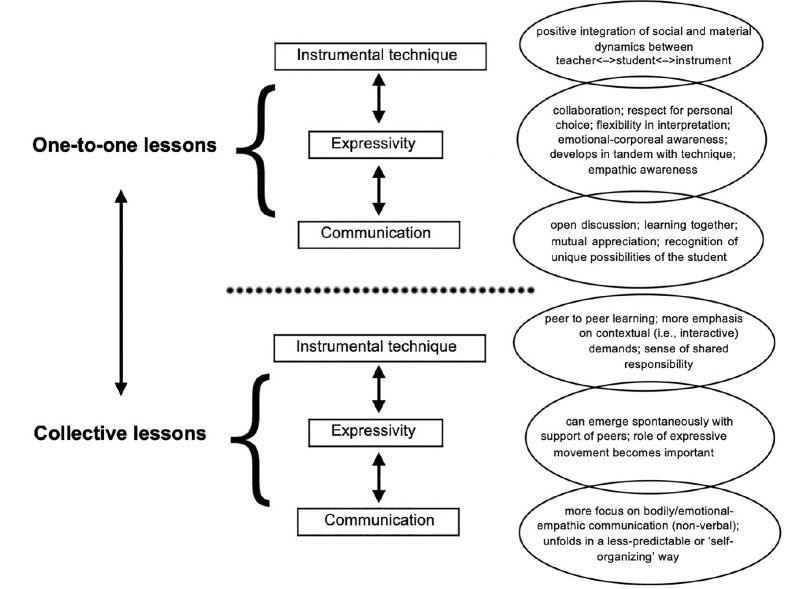
The students’ reports on the dynamics of positive learning environments in one-to-one and collective contexts. It shows the overlapping nature of the coding categories – instrumental technique, expressivity, and communication – in both contexts.

Our findings (summarized in Figure 2), align with the emerging approaches to music pedagogy and embodied cognition discussed at the outset of this paper. As recent work in music education trades the traditional focus on the score, individualized technical aspects, and strict rules of interpretation and practice for processes that allow the collaborative and creative constitution of the musical event in a broader sense, so ECS trades the focus on the functional rules of states that are instantiated in the brain for the embodied, ecological, and relational processes that are constitutive to the broader realization of mental life. The latter, understood as an integrated brain-body-world system, entails sensorimotor forms of engagement with the socio-material environment that provide an agent with the necessary configuration to act, think, and learn in ways that are meaningful. The reported statements indicated that students have positive experiences when musical learning involves cooperation – when they are free to explore musical possibilities with teachers and peers. This also indicates that for effective musical development to happen, the technical aspects of musical learning cannot be separated from those associated with expressivity and communication – with the contingencies of musical activity as it plays out in living social environments. Because ECS appears to provide ways of better understanding such interactive environments, we suggest that it offers useful insights that can contribute to research, theory, and practice in music education.

## Author Contributions

AS, DvdS, MB, and RP designed the study. AS, DvdS, and MB collected the data. AS, MB, and NM analyzed the data. AS, DvdS, and NM wrote the manuscript. MB and RP provided comments and suggestions that were implemented in the final draft. All authors approved the final version of the manuscript.

## Conflict of Interest Statement

The authors declare that the research was conducted in the absence of any commercial or financial relationships that could be construed as a potential conflict of interest.

## Footnotes

1 While many theorists argue that there is no syntax without semantics, others suggest that it may be possible to have computational operations on non-interpreted symbols. When syntactic operations mirror semantics, then semantics are obtained “for free.” As Haugeland once famously put it “take care of syntax, and semantics will take care of itself.”
